# Friendship Importance Around the World: Links to Cultural Factors, Health, and Well-Being

**DOI:** 10.3389/fpsyg.2020.570839

**Published:** 2021-01-18

**Authors:** Peiqi Lu, Jeewon Oh, Katelin E. Leahy, William J. Chopik

**Affiliations:** ^1^Department of Counseling and Clinical Psychology, Columbia University, New York, NY, United States; ^2^Department of Psychology, Michigan State University, East Lansing, MI, United States

**Keywords:** friendship, collectivism/individualism, Hofstede’s cultural dimensions, health, happiness, World Values Survey (WVS)

## Abstract

Prioritizing friendship is associated with many health and well-being benefits. However, to date, there have been relatively few studies that have examined cultural moderators of the link between friendship and important outcomes. In other words, is prioritizing friendships more beneficial in some contexts than others? In the current study, we examined how culture- and country-level factors were associated with the importance people place on friendships and the benefits derived from this importance. The sample comprised of 323,200 participants (*M* = 40.79 years, *SD* = 16.09 years) from 99 countries from the World Values Survey. Multilevel analyses revealed that women, people with higher levels of education, and people living in countries that are more economically equal and high in indulgence placed more value on friendships. Prioritizing friendships in life was associated with better health and well-being, but these associations depended on many cultural factors. The findings are discussed in the context of the ways in which friendships can enrich health and well-being across different settings.

## Introduction

Friendships enrich our lives in many ways. Friends give us both practical and emotional support when we need it. As a result, there are many emotional and physical health benefits of friendships—the more people prioritize friendships, the happier and healthier they are. Moreover, broader cultural contexts can have large influences on how friendships function and are expressed. Therefore, the benefits that people accrue from friendships might also vary across cultures. In the current study, we examined how the importance people place on friendships varies across cultures and whether this variation is associated with differences in the health and well-being of the people living in those cultures.

### The Role of Friendship in Health and Happiness

There is a reliable link between social support and mental and physical health across the lifespan (Holt-Lunstad et al., 2010, 2015; Holt-Lunstad, 2017), and one important source of support is our friends. Friends provide us with a strong sense of companionship, mitigate feelings of loneliness (Lykes and Kemmelmeier, 2014), and contribute to our self-esteem and life satisfaction (Goodwin and Hernandez Plaza, 2000; Chopik, 2017). Perceiving greater support from friends is associated with a greater sense of purpose and control over one’s life (Veiel and Baumann, 1992; Leary et al., 1995). In terms of predicting health, friendship occasionally predicts health to an equivalent and, in some cases, larger degree compared to spousal and parent–child relationships (Bearman and Moody, 2004; Giles et al., 2005; Christakis and Fowler, 2007). Friends also help individuals institute healthy behaviors in their own lives. For example, seeing a friend trying to lose weight is associated with an individual’s commitment to maintaining a healthy weight (Wing and Jeffery, 1999; Gorin et al., 2005).

However, friendship is not universally good for individuals—depression and negative health behaviors can also spread through friend networks (Smith and Christakis, 2008; Rosenquist et al., 2011). For instance, the risk of obesity, suicide, smoking, and other forms of substance abuse increase dramatically when surrounded by peers who are obese and/or suicidal, smoke, and abuse substances (Urberg et al., 1997; Andrews, 2002; Bearman and Moody, 2004; Christakis and Fowler, 2007). In sum, friends play a significant role in people’s mental and physical well-being, for better and for worse. Nevertheless, the degree to which people value and benefit from friendship may differ across settings and cultures. In other words, different country-level factors might predict how much people value friendships and, in turn, the benefits that people obtain from friendships.

### Do Friendships Vary Across Countries?

Some form of friendship is present in nearly all cultures and countries (Cohen, 1966), but friendships are perceived and constructed differently across cultures (Baumgarte, 2016). While some cultures employ a looser definition of friendship, others are stricter in the ways they define friendship (Stewart and Bennett, 1991; Goodwin, 1999). Based on how people define friendship, there is accompanying variance in how many friends people have and what people expect from friends. For instance, a cross-national study in friendship found that Americans were more likely to have more friends and differentiate between friends; Ghanaians were more cautious toward friends and having a large group of friends (Adams and Plaut, 2003). Likewise, people’s understanding of intimacy in friendship varies across cultures (Keller, 2004a). Compared to Chinese adolescents, Western adolescents emphasize more on relationship intimacy and quality interactions in their friendships (Keller et al., 1998; Keller, 2004b). In addition, friendships are more stable and fixed in some societies and more flexible and relationships of choice in other societies. In the latter case, relationships can change more rapidly as people have the freedom to voluntarily choose relationships (i.e., higher relational mobility). As a result, people tend to trust strangers more and are more proactive in maintaining friends, self-disclosing, and provide more support (Schug et al., 2010; Thomson et al., 2018). These behaviors are characteristic of friendships in individualistic cultures, as individualistic cultures possess higher relational mobility (Kito et al., 2017).

There are also several country-level (e.g., gross domestic product) and individual factors (e.g., gender) that might explain differences in how people define and value friendships and the benefits that people accrue from friendships. However, there have been almost no large-scale examinations of cross-cultural differences in friendship processes. As a result, in the present study, we took a largely exploratory approach to how country-level factors might alter whether people value friendships in their lives. Research has established different dimensions for social and cultural constructs. One such framework is Hofstede’s cultural dimensions, which consists of six national constructs through which countries organize themselves (Hofstede et al., 2010). Although there are many dimensions on which cultures vary, we elected to focus primarily on the Hofstede dimensions given the great breadth of research on their links to health, well-being, and social behavior and characteristics identified in past research. However, we do run some supplementary analyses examining other taxonomies of cultural dimensions.

Below, we briefly discuss the concept of friendship importance and ways in which friendships might vary across country-level factors that have been traditionally studied by researchers (e.g., gross domestic product, income inequality, Hofstede’s cultural dimensions) and how these factors might influence the effects of friendships on health and well-being. Specifically, for each factor, we review its links with well-being and speculate how it might influence friendship importance and interact with friendship importance to predict well-being.

#### Friendship Importance

Previous studies have found that friends are important for personal well-being. However, there is little research that explicitly explores the effect of *valuing* friendship on important life outcomes, like well-being and life satisfaction. Instead, friendship researchers have examined the number of friends (Ho, 2016), quality of friendship (Demir et al., 2012), best friends (Demir and Özdemir, 2010), and support from friends (Secor et al., 2017). However, there is variation in how people define friendship, define closeness and support, and define what kind of friends they might have (Miche et al., 2013; Baumgarte, 2016). Our study utilizes a different way of thinking about friendship—how much people value friendship (i.e., friendship importance). Values direct people’s thoughts and behaviors toward efforts that they consider important (Kluckhohn, 1951; Rokeach, 1973; Schwartz, 1992), so this broad measure may capture people having good friends, receiving and giving social support to friends, and interacting with friends, but is not so specific that it would confuse people from different cultures.

People might devote more resources to their friends and have higher quality relationships if they value friends and find them important (Roberts et al., 2005). Or people might find friends important *because* they have high quality relationship and their friends hold vital roles in their lives. Indeed, there is some evidence that when friendships are evaluated as important, people experience well-being benefits. For example, feeling committed to the role of being a friend is positively related to life satisfaction, even when controlling for ostensibly more detailed measures of social network involvement (e.g., support network density; Siebert et al., 1999). Thus, we are treating friendship importance—the degree to which people find friends important and value them—as a proxy for how much people investing in friendships and likely how good friendships are. However, we do acknowledge that specificity is lost in this trade-off for an increased understanding of the instrument across cultural settings. Although there are relatively few studies that investigated how valuing friendship might influence their behaviors and important life outcomes, a previous study using the World Values Survey found that friendship importance predicted better health and happiness while controlling for family importance (Chopik, 2017). However, several country-level factors might predict how people value friendship and the extent to which friendship importance is associated with health and well-being. In the sections below, we provide a short introduction to the country-level factors that we focused on in the current study.

#### Gross Domestic Product

Gross domestic product (GDP) reflects a country’s economic status, and richer societies often have a higher GDP. With improvements in national GDP, citizens benefit from decreased child labor, lower rates of unemployment, increased school attendance, upgrades in transportation and healthcare services, and other improvements in infrastructure (Moniruzzaman and Andersson, 2008; Muazzam and Nasrullah, 2011). Greater GDP is associated with country-level health indicators, including reductions in child and all-cause mortality rates (Ward and Viner, 2017), as well as increases in the amount and variety of opportunities for individuals to attain their personal goals and pursue their interests (Clark and Senik, 2011). Importantly, GDP is positively associated with life engagement, one of the indicators of subjective well-being (Hill et al., 2019). On the one hand, because *lower* GDP often portends several life difficulties (e.g., health, quality of life of individuals from low-GDP countries may have more stressful relationships—an association often seen at the individual level with socioeconomic status; Veenstra, 2000). On the other hand, social networks are a protective factor against stress for people living in low- and middle-income countries (Perkins et al., 2015). Therefore, we expect that lower GDP might be associated with people valuing friendships less. However, among people who do value friendship, lower GDP might have a less negative impact on life outcomes because valuing friendships might offset the negative effects of local economic conditions.

#### Income Inequality

The GINI index of income inequality measures a nation’s unequal distribution of wealth among its citizens (Central Intelligence Agency, 2011). Overall, quality of life is higher in countries with lower levels of inequality: people are happier, more satisfied, and report greater purpose in life (Oishi et al., 2011; Pickett and Wilkinson, 2015; Cheung, 2018; Hill et al., 2019). Income inequality is associated with increased all-cause and communicable disease mortality (Ward and Viner, 2017). It may be that, in unequal societies where differences in social status, power, and wealth are more prominent and many social relations are vertical, people value horizontal relationships like friendship more for its focus on reciprocity and sharing (Wilkinson, 1999). Alternatively, it could be the case that societies with more inequality value friendships less—the salient financial inequality might alter the things that people value in their lives (e.g., they might think it is more important to spend their time working harder to get ahead or meet people with a higher status rather than spend time with peers and friends). Nonetheless, like GDP, even under high income inequality, valuing friendships might buffer against the negative effects of income inequality on health and well-being via the benefits that people receive from friends’ support (Wilkinson, 1999; Perkins et al., 2015).

#### Power Distance

The extent to which individuals with less power accept inequalities in control and influence, defined as a country’s power distance index (PDI), is associated with subjective well-being at a national level (Ye et al., 2015; Karibayeva and Kunanbayeva, 2018). In close relationships, power differences between relational partners oftentimes predict commitment to a relationship, how they make decisions in various domains, and how they express dominance behaviors while interacting with each other (Dunbar and Burgoon, 2005; Farrell et al., 2015). However, it is unclear whether PDI would predict how much people value friendships and whether PDI enhances or diminishing the positive effects of valuing friendships.

#### Individualism/Collectivism

Individualistic countries prefer the preservation and championing of individual freedoms and more diffuse social networks; collectivist countries prefer closely bonded social (often familial) networks and interpersonal harmony (Hofstede, 1984; Triandis, 1995; Keller et al., 1998). As a result, people from individualistic cultures rated the lack of interaction with friends as their main source of loneliness, and people from collectivist cultures rated the poor quality of familial relationships and communication as the main sources of their loneliness (Lykes and Kemmelmeier, 2014). However, this is not to say that collectivism would be linked with lower friendship importance. People from individualistic cultures tend to report having more friends, show less caution toward friends, and feel sorry for those without friends, which might imply a positive association between individualism and valuing friendships (Adams, 2005). Although individualism/collectivism has been the most extensively studied cultural factor in friendship research, the number of studies is still small and these studies occasionally find no differences between individualistic and collectivistic cultures, especially after childhood (e.g., Keller et al., 1998). Further, research to date often compares how friendship processes differ between only two countries, ignoring the diversity of individualism/collectivism across other countries around the world and other factors beyond individualism/collectivism that might account for differences between two countries. Therefore, in the current study, we explore several countries that vary across the individualism/collectivism spectrum and examine its influence on the degree to which people value friendship and benefit from doing so.

#### Masculinity vs. Femininity

Masculinity corresponds to being more assertive, more interested in the acquisition of status and resources, and a lower focus on the care and affection of others (Holleran et al., 1988). Assertiveness is a social skill that allows people to communicate directly with others about their desires (Arrindell and Van der Ende, 1985) and indirectly leads to increases in subjective well-being and general positivity (Argyle and Lu, 1990; Lauriola and Iani, 2017). However, assertiveness and masculinity are not *exclusively* beneficial for people’s health and well-being. For example, self-reliance and independence are associated with fewer medical checkups, which may translate into poorer health outcomes (Calasanti, 2004; Springer and Mouzon, 2011). In the context of friendship, people in masculine societies might be more self-reliant and do not depend on or value friendships as much. Because there have been no large-scale comparisons of friendship processes between more masculine and feminine cultures, we did not make formal hypotheses about how masculinity/femininity would be associated with the importance people place on friendship.

#### Uncertainty Avoidance

Uncertainty and ambiguity in situations can be a source of stress and threat that impede people’s well-being (Roll et al., 2015). At the country level, the Uncertainty Avoidance Index (UAI) describes a country’s intolerance for uncertainty and instability (Hofstede, 1984). Societies that tend to avoid uncertainty are characterized by more anxiety and aggression aimed at achieving stability and predictability in their society. On the one hand, higher levels of uncertainty avoidance may be associated with lower levels of health, happiness, and well-being due to countries having characteristically higher levels of anxiety (Voshaar et al., 2015). Because friends provide support for individuals, valuing friendships may alleviate concerns about uncertainty by leading people to seek support from friends that may provide some certainty (Hitlin and Piliavin, 2004). Therefore, high uncertainty avoidance may be associated with valuing friendships because they serve this comforting role. On the other hand, uncertainty avoidance could motivate a society toward investing in solutions and policies that introduce predictability and ultimately enrich people’s lives rather than leaving its citizens to be comforted by members of their social network. As a result, friendships might not be particularly important for or linked with UAI.

#### Long-Term Orientation

Long-term orientation refers to the set of beliefs and behaviors aimed at cultivating long-term desirable outcomes (Hofstede, 2001). For example, people who endorse a long-term orientation are more willing to sacrifice current satisfaction and pleasure for long-term outcomes (O’Keefe, 2002). Several studies suggest that resistance to consumption and valuing long-term goals lead to greater well-being among individuals and more sustainable societies (Sheth et al., 2011; Chua et al., 2015). In addition, people are usually more willing to sacrifice for and cooperate with their friends when they expect reciprocity from their friends in the future (Van Lange et al., 1997; Van Lange and Joireman, 2008). Maintaining long-term committed relationships strengthens physical and psychological well-being (Dush and Amato, 2005; Loving and Slatcher, 2013). Given the long-term benefits of friendships, we might expect long-term orientation to be linked with placing higher importance in friendships.

#### Indulgence vs. Restraint

Indulgence refers to the extent to which societies allow for the gratification of basic and natural human desires (Hofstede, 2011). A more indulgent society allows for free expression and engagement in these desires; a more restrained society imposes social norms as a means to restrict the gratification of these desires. Research in marketing suggests that indulgent consumption is one source from which people derive pleasure and happiness (Haws and Poynor, 2008; Hagtvedt and Patrick, 2009). On the other hand, indulgence sometimes activates negative emotions, such as guilt and regret (Kivetz and Simonson, 2002; Keinan et al., 2016). A country high in indulgence may encourage individuals to engage in pleasurable activities, which would result in reduced stress and better health (Petersen et al., 2018). To our knowledge, no research to date has examined indulgence versus restraint predicting friendship characteristics. However, individuals who feel free to engage in pleasurable activities (i.e., in an indulgent society), like spending time with friends, might value and even benefit from friendships more.

## The Current Study

The current study assessed the importance people place on friendships, health, happiness, and subjective well-being in a sample of 323,200 participants from 99 countries. We focused on two questions: first, which individual- and country-level factors are associated with variation in friendship importance across countries? Second, what individual and country-level factors might interact with friendship importance to predict health and well-being? Is valuing friendships particularly beneficial in some countries compared to others? Many of our questions were exploratory—little research existed to guide our hypotheses beyond a select few studies examining differences between individualistic and collectivistic countries and comparing two countries (Keller et al., 1998; Kito, 2005; Lykes and Kemmelmeier, 2014; Baumgarte, 2016). The results from the current study can shed light on how cultural contexts affect friendships and the benefits that individuals accrue from them.

## Materials and Methods

### Participants and Procedure

**FIGURE 1:**
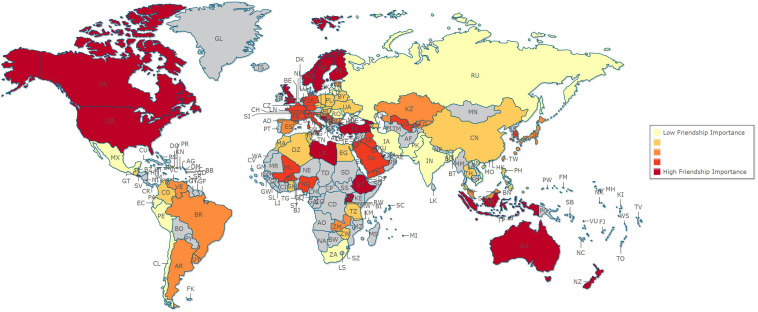
Ratings of friendship importance from 99 countries.

Participants were 323,200 individuals (51.7% female) from the World Values Survey (WVS; see Inglehart et al., 2008). Since 1981, the WVS has interviewed representative national samples of several different countries all around the world. Information on publications, findings, methodology, and free data access are available at http://www.worldvaluessurvey.org. For the current study, data from waves 1 to 5 of the WVS were aggregated, and 99 different countries are represented in the current report (see Figure 1 for country coverage). Sample sizes ranged from 400 (Dominican Republic) to 15,088 (South Africa), with an average sample size of 3,265 (*SD* = 2,479). The overall sample ranged in age from 15 to 99 (*M* = 40.79 years, *SD* = 16.09 years); the median level of education was some secondary education. Each decade of life was well represented (e.g., 15–19 years: 17,139; 20–29 years: 79,948; 30–39 years: 71,689; 40–49 years: 59,919; 50–59 years: 44,318; 60–69 years: 30,889; 70 + years: 19,298); consistent demographic information on participants across cultures was limited to age, gender, and education.

Because we analyzed an existing data source, the Michigan State Institutional Review Board considered this research exempt from ethical oversight, as it did not constitute human subjects research (IRB# STUDY00002967).

### Measures

#### Country-Level Characteristics

Country-level GDP per capita (*n* = 93 countries had available data; Central Intelligence Agency, 2011) and the GINI index of income inequality (*n* = 88 countries had available data; Central Intelligence Agency, 2011) were gathered as country-level characteristics that measure the economic conditions of a country.

Hofstede’s dimensions of cultural variation were also included in the analysis. Hofstede et al. (2010) suggest that country-level differences in societal values can be characterized by six dimensions.

*Power Distance (PDI)* measures the degree to which a culture is accepting of inequality. *Individualism/collectivism (IDV)* refers to the degree to which people prefer loosely knit social networks and individuality (individualism; higher values) versus tightly knit social networks and interdependence with others (collectivism; lower values). *Masculinity/Femininity (MAS)* assesses the degree to which a culture can be characterized by assertiveness and competitiveness (masculinity; higher values) or nurturance and cooperation (femininity; lower scores). *Uncertainty Avoidance (UAI)* measures the degree to which a country’s citizens are uncomfortable with uncertainty and ambiguity. *Long-Term Orientation (LTO)* assesses the outlook of a culture; countries with a long-term orientation place more importance on the future. *Indulgence vs. restraint (IVR)* refers to the degree to which a society allows free gratification of basic and natural human drives related to enjoyment of life (relative to a suppression of gratification of needs by strict social norms).

Scores on each of these dimensions were gathered from Hofstede’s latest reporting on cultural dimensions (Hofstede et al., 2010). Country-level scores on all of the dimensions were available for 57 countries in the current analyses (and for a total of 83 and 85 countries for long-term orientation and indulgence vs. restraint, respectively).

#### Friendship Importance

Participants were asked to indicate how important friends were in their lives on a scale ranging from 1 (*very important*) to 4 (*not at all important*). Scores were recoded such that higher values reflected more importance placed on friendships. Worth noting, participants were asked about relational values only in waves 2–5.^1^

#### Self-Rated Health

Health was assessed at each wave with a single item, “All in all, how would you describe your state of health these days?” Participants rated their health on a scale ranging from 1 (*very good*) to 4 (*poor*). Responses were reverse-scored so that higher values reflected better self-rated health. Numerous studies have shown that self-rated health measures are strong predictors of mortality (Idler and Benyamini, 1997; Schnittker and Bacak, 2014).

#### Happiness and Subjective Well-Being

Happiness was measured with a single item, “Taking all things together, would you say you are…” Participants rated their happiness on a scale ranging from 1 (*very happy*) to 4 (*not at all happy*). Responses were reverse scored so that higher values reflected more happiness. Subjective well-being was measured with a single item, “All things considered, how satisfied are you with your life as a whole these days?” Participants responded to this item on a 10-point scale ranging from 1 (*completely dissatisfied*) to 10 (*completely satisfied*).

## Results

### What Is Associated With Variation in Friendship Importance Across Countries?

Because respondents were nested within countries, a multilevel model predicting friendship importance was created, using the SPSS MIXED procedure (Peugh and Enders, 2005). Participant age, participant gender (−1 = male, 1 = female), education, and country-level variables (i.e., GDP, GINI, PDI, IDV, MAS, UAI, LTO, IVR; see table notes) were entered as predictors of friendship importance across countries.^2^ All continuous individual and country-level variables were grand-mean centered for these analyses. The country-level standing on friendship importance can be seen in Figure 1. Results from this multilevel model are presented in Table 1. Older adults valued friendship less compared to younger adults. Women, people with higher levels of education, and people from countries low in inequality and high in indulgence placed higher importance on friendship in their lives. GDP, power distance, individualism/collectivism, masculinity, uncertainty avoidance, and long-term orientation did not significantly predict friendship importance.

**TABLE 1:** Multilevel models predicting friendship values.

					95% CI		
	*b*	*SE*	*t*	*p*	*LB*	*UB*	*r*
Intercept	3.28	0.03	119.97	<0.001	3.22	3.33	
Age	–0.002	0.0001	–16.31	<0.001	–0.002	–0.002	–0.037
Gender	0.01	0.002	3.60	<0.001	0.003	0.01	0.008
Education	0.03	0.001	40.83	<0.001	0.03	0.03	0.093
GDP	0.02	0.02	0.79	0.43	–0.02	0.05	0.122
GINI	–0.01	0.004	–2.05	0.05	–0.02	–0.0001	–0.305
PDI	–0.0003	0.002	–0.18	0.86	–0.004	0.004	–0.027
IDV	0.001	0.002	0.87	0.39	–0.002	0.005	–0.135
MAS	–0.001	0.001	–0.70	0.49	–0.004	0.002	–0.108
UAI	–0.001	0.001	–0.83	0.41	–0.003	0.001	–0.128
LTO	0.002	0.001	1.38	0.18	–0.001	0.01	0.210
IVR	0.005	0.001	3.16	0.003	0.002	0.01	0.442

*Gender: -1, male; 1, female; GDP, gross domestic product; GINI, income inequality; PDI, power distance index; IDV, individualism (higher values); MAS, masculinity (higher values); UAI, uncertainty avoidance index; LTO, long-term orientation; IVR, indulgence/restraint. Continuous variables were grand-mean centered.*

### Do Individual- and Country-Level Constructs Moderate the Association Between Friendship Importance and Health, Happiness, and Subjective Well-Being?^3^

Because respondents were nested within countries, three multilevel models (for health, happiness, and subjective well-being) were created, using the SPSS MIXED procedure (Peugh and Enders, 2005). Participant age, participant gender (−1 = male, 1 = female), friendship importance, education, and country-level variables (i.e., GDP, GINI, PDI, IDV, MAS, UAI, LTO, IVR) were entered as predictors of each outcome across countries.^4^ Further, all possible interactions between individual- and country-level variables with friendship importance were also modeled. All continuous individual and country-level variables were grand-mean centered for these analyses.

Results from these multilevel models are presented in Table 2 (for health), Table 3 (for happiness), and Table 4 (for subjective well-being).

**TABLE 2:** Multilevel models predicting health.

					95% CI		
	*b*	*SE*	*t*	*p*	*LB*	*UB*	*r*
Intercept	3.85	0.03	139.47	<0.001	3.79	3.90	
Age	–0.01	0.0001	–104.83	<0.001	–0.01	–0.01	–0.238
Gender	–0.04	0.002	–22.64	<0.001	–0.05	–0.04	–0.053
Education	0.05	0.0009	55.60	<0.001	0.05	0.05	0.129
Friendship	0.10	0.003	32.95	<0.001	0.10	0.11	0.077
GDP	0.04	0.02	2.03	0.05	0.0001	0.08	0.302
GINI	–0.002	0.004	–0.50	0.62	–0.009	0.006	–0.078
PDI	–0.004	0.002	–1.81	0.08	–0.007	0.0004	–0.271
IDV	–0.001	0.002	–0.85	0.40	–0.005	0.002	–0.131
MAS	0.0003	0.001	0.18	0.86	–0.003	0.003	0.028
UAI	–0.003	0.001	–2.38	0.02	–0.005	–0.0004	–0.348
LTO	0.003	0.002	–1.91	0.06	–0.006	0.0001	–0.286
IVR	0.003	0.002	2.25	0.03	0.0004	0.006	0.332
Age × Friendship	0.001	0.0002	3.73	<0.001	0.0003	0.001	0.009
Gender × Friendship	0.01	0.003	3.02	0.003	0.003	0.01	0.007
Education × Friendship	–0.004	0.001	–3.43	0.001	–0.01	–0.002	–0.008
GDP × Friendship	–0.003	0.002	–1.48	0.14	–0.01	0.001	–0.003
GINI × Friendship	–0.0003	0.0003	–1.05	0.29	–0.001	0.0003	–0.002
PDI × Friendship	0.001	0.0002	2.21	0.03	0.0001	0.001	0.005
IDV × Friendship	0.001	0.0002	5.90	<0.001	0.001	0.001	0.014
MAS × Friendship	0.001	0.0002	2.74	0.01	0.0001	0.001	0.006
UAI × Friendship	0.00003	0.0001	0.24	0.81	–0.0002	0.0003	0.001
LTO × Friendship	0.0003	0.0002	1.96	0.05	0.000001	0.001	0.005
IVR × Friendship	–0.0002	0.0002	–1.26	0.21	–0.001	0.0001	–0.003

*Gender: -1, male; 1, female; GDP, gross domestic product; GINI, income inequality; PDI, power distance index; IDV, individualism (higher values); MAS, masculinity (higher values); UAI, uncertainty avoidance index; LTO, long-term orientation; IVR, indulgence/restraint. Continuous variables were grand-mean centered.*

**TABLE 3:** Multilevel models predicting happiness.

					95% CI		
	*b*	*SE*	*t*	*p*	*LB*	*UB*	*r*
Intercept	3.11	0.02	167.92	<0.001	3.07	3.15	
Age	–0.002	0.0001	–14.65	<0.001	–0.002	–0.001	–0.034
Gender	0.02	0.002	10.58	<0.001	0.014	0.02	0.025
Education	0.02	0.0008	28.72	<0.001	0.10	0.02	0.067
Friendship	0.10	0.003	38.22	<0.001	0.02	0.11	0.088
GDP	0.03	0.01	2.31	0.03	0.004	0.06	0.339
GINI	–0.004	0.002	–1.46	0.15	–0.008	0.001	–0.223
PDI	–0.002	0.001	–1.27	0.21	–0.004	0.001	–0.194
IDV	–0.003	0.001	–2.57	0.01	–0.005	–0.0006	–0.372
MAS	0.0009	0.0009	0.94	0.36	–0.001	0.003	0.144
UAI	–0.003	0.0008	–3.74	0.001	–0.005	–0.001	–0.505
LTO	–0.002	0.001	–2.13	0.04	–0.004	–0.0001	–0.316
IVR	0.006	0.001	6.04	<0.001	0.004	0.008	0.686
Age × Friendship	0.001	0.0001	5.40	<0.001	0.0005	0.001	0.013
Gender × Friendship	0.01	0.002	3.90	<0.001	0.004	0.01	0.009
Education × Friendship	–0.01	0.001	–5.61	<0.001	–0.01	–0.004	–0.013
GDP × Friendship	–0.001	0.002	–0.47	0.64	–0.005	0.003	–0.001
GINI × Friendship	0.0001	0.0003	0.20	0.84	–0.0005	0.001	0.000
PDI × Friendship	0.001	0.0002	2.83	0.01	0.0002	0.001	0.007
IDV × Friendship	0.001	0.0002	4.53	<0.001	0.0004	0.001	0.011
MAS × Friendship	0.003	0.0002	1.94	0.05	–0.000004	0.001	0.005
UAI × Friendship	–0.0001	0.0001	–1.03	0.31	–0.0004	0.0001	–0.002
LTO × Friendship	0.0002	0.0001	1.14	0.25	–0.0001	0.0004	0.003
IVR × Friendship	0.0001	0.0001	0.61	0.55	–0.0002	0.0004	0.001

*Gender: -1, male; 1, female; GDP, gross domestic product; GINI, income inequality; PDI, power distance index; IDV, individualism (higher values); MAS, masculinity (higher values); UAI, uncertainty avoidance index; LTO, long-term orientation; IVR, indulgence/restraint. Continuous variables were grand-mean centered.*

**TABLE 4:** Multilevel models predicting subjective well-being.

					95% CI		
	*b*	*SE*	*t*	*p*	*LB*	*UB*	*r*
Intercept	6.83	0.06	124.09	<0.001	6.72	6.94	
Age	0.001	0.0003	3.21	<0.001	0.0004	0.002	0.007
Gender	0.04	0.005	7.20	<0.001	0.03	0.05	0.017
Education	0.10	0.002	40.17	<0.001	0.09	0.10	0.093
Friendship	0.25	0.008	29.51	<0.001	0.23	0.27	0.068
GDP	0.14	0.04	3.48	0.001	0.06	0.22	0.477
GINI	–0.02	0.007	–2.94	0.005	–0.04	–0.007	–0.418
PDI	–0.01	0.004	–2.91	0.006	–0.02	–0.003	–0.413
IDV	–0.02	0.003	–4.47	<0.001	–0.02	–0.008	–0.572
MAS	0.004	0.003	1.41	0.17	–0.002	0.01	0.215
UAI	–0.005	0.002	–2.24	0.03	–0.01	–0.0005	–0.331
LTO	–0.006	0.003	–2.11	0.04	–0.01	–0.0003	–0.314
IVR	0.03	0.003	8.64	<0.001	0.02	0.03	0.803
Age × Friendship	0.001	0.0004	2.65	0.01	0.0003	0.002	0.006
Gender × Friendship	0.02	0.01	3.68	<0.001	0.01	0.04	0.009
Education × Friendship	–0.02	0.003	–5.79	<0.001	–0.03	–0.01	–0.013
GDP × Friendship	–0.01	0.01	–1.85	0.07	–0.02	0.001	–0.004
GINI × Friendship	0.002	0.001	2.26	0.02	0.0002	0.003	0.005
PDI × Friendship	–0.001	0.001	–1.35	0.18	–0.002	0.0004	–0.003
IDV × Friendship	0.003	0.001	5.20	<0.001	0.002	0.003	0.012
MAS × Friendship	0.0004	0.001	0.76	0.45	–0.001	0.001	0.002
UAI × Friendship	0.001	0.0004	3.09	0.002	0.0004	0.002	0.007
LTO × Friendship	0.002	0.0004	3.52	<0.001	0.001	0.002	0.008
IVR × Friendship	–0.002	0.0004	–4.80	<0.001	–0.003	–0.001	–0.011

*Gender: -1, male; 1, female; GDP, gross domestic product; GINI, income inequality; PDI, power distance index; IDV, individualism (higher values); MAS, masculinity (higher values); UAI, uncertainty avoidance index; LTO, long-term orientation; IVR, indulgence/restraint. Continuous variables were grand-mean centered.*

#### Health

Valuing friendship was associated with better health across cultures (see Table 2). People reported worse health if they were older, women, less educated, and from countries lower in GDP, lower in indulgence, and higher in uncertainty avoidance.

There were many instances in which the link between valuing friendship and health was moderated by individual- or country-level variables. Specifically, there were significant two-way interactions between friendship importance and age, gender, education, power distance, individualism, masculinity, and long-term orientation. The simple slopes of each of these effects on health at high (+1 *SD*) and low friendship importance (−1 *SD*) are presented in Table 5. Friendship importance was more strongly related to health among older adults, women, people with less education, and people from countries higher in power distance, individualism, femininity, and long-term orientation.

**TABLE 5:** Analyses decomposing the effect of friendship importance at 1 SD above and below the mean of a moderator.

	-1 Standard deviation on variable (or men)							+1 Standard deviation on variable (or women)						
					95% CI							95% CI		
	*b*	*SE*	*t*	*p*	*LB*	*UB*	*r*	*b*	*SE*	*t*	*p*	*LB*	*UB*	*r*
**Health**														
Age	0.092	0.004	22.080	<0.001	0.084	0.100	0.051	0.112	0.004	28.637	<0.001	0.104	0.119	0.067
Gender	0.098	0.004	22.190	<0.001	0.090	0.107	0.074	0.104	0.004	23.778	<0.001	0.095	0.112	0.077
Education	0.111	0.004	28.858	<0.001	0.103	0.118	0.067	0.093	0.004	22.090	<0.001	0.085	0.101	0.051
PDI	0.093	0.005	18.591	<0.001	0.084	0.103	0.043	0.111	0.005	22.672	<0.001	0.101	0.120	0.053
IDV	0.078	0.005	15.062	<0.001	0.068	0.088	0.035	0.126	0.005	25.162	<0.001	0.116	0.136	0.059
MAS	0.093	0.004	23.940	<0.001	0.086	0.101	0.056	0.111	0.005	22.364	<0.001	0.101	0.120	0.052
LTO	0.094	0.005	18.476	<0.001	0.084	0.104	0.043	0.110	0.005	20.717	<0.001	0.100	0.121	0.048
**Happiness**														
Age	0.090	0.004	24.972	<0.001	0.083	0.098	0.058	0.115	0.003	33.825	<0.001	0.108	0.121	0.078
Gender	0.096	0.004	24.886	<0.001	0.089	0.104	0.082	0.109	0.004	29.037	<0.001	0.102	0.116	0.094
Education	0.022	0.001	28.723	<0.001	0.021	0.024	0.067	0.090	0.004	24.558	<0.001	0.083	0.097	0.057
PDI	0.093	0.004	21.334	<0.001	0.085	0.102	0.050	0.112	0.004	26.454	<0.001	0.104	0.120	0.061
IDV	0.087	0.004	19.520	<0.001	0.078	0.095	0.045	0.118	0.004	27.156	<0.001	0.110	0.127	0.063
**Subjective Well-being**														
Age	0.231	0.011	20.241	<0.001	0.208	0.253	0.047	0.268	0.011	25.153	<0.001	0.248	0.289	0.058
Gender	0.232	0.012	19.138	<0.001	0.208	0.256	0.063	0.271	0.012	22.782	<0.001	0.247	0.294	0.074
Education	0.291	0.010	27.672	<0.001	0.270	0.311	0.064	0.209	0.012	18.118	<0.001	0.186	0.231	0.042
GINI	0.232	0.013	17.884	<0.001	0.206	0.257	0.041	0.267	0.010	26.685	<0.001	0.248	0.287	0.062
IDV	0.192	0.014	13.584	<0.001	0.164	0.220	0.031	0.307	0.014	22.414	<0.001	0.280	0.334	0.052
UAI	0.225	0.012	18.536	<0.001	0.201	0.249	0.043	0.274	0.011	24.880	<0.001	0.253	0.296	0.058
IVR	0.301	0.014	21.871	<0.001	0.274	0.328	0.051	0.198	0.014	14.558	<0.001	0.171	0.225	0.034

*GDP, gross domestic product; GINI, income inequality; PDI, power distance index; IDV, individualism (higher values); MAS, masculinity (higher values); UAI, uncertainty avoidance index; LTO, long-term orientation; IVR, indulgence/restraint.*

#### Happiness

Valuing friendship was associated with greater happiness across cultures (see Table 3). People reported lower happiness if they were older, male, less educated, and from countries lower in GDP, higher in individualism, higher in uncertainty avoidance, more restrained, and higher in long-term orientation.

There were many instances in which the effects of friendship importance on happiness were moderated by individual- or country-level variables. Specifically, there were significant two-way interactions between friendship importance and age, gender, education, power distance, and individualism. The simple slopes of each of these effects on happiness at high (+1 *SD*) and low friendship importance (−1 *SD*) are presented in Table 5. Friendship importance was more strongly related to happiness among older adults, women, people with less education, and people from countries higher in power distance and individualism.

#### Subjective Well-Being

Valuing friendship was associated with higher levels of subjective well-being across cultures (see Table 4). People reported lower subjective well-being if they were younger, male, less educated, and from countries lower in GDP, higher in inequality, higher in power distance, higher in individualism, higher in uncertainty avoidance, and higher in long-term orientation.

There were many instances in which the effects of friendship importance on subjective well-being were moderated by individual- or country-level variables. Specifically, there were significant two-way interactions between friendship importance and age, gender, education, inequality, individualism, uncertainty avoidance, long-term orientation, and indulgence. The simple slopes of each of these effects on subjective well-being at high (+1 *SD*) and low friendship importance (−1 *SD*) are presented in Table 5. Friendship importance was more strongly related to subjective well-being among older adults, women, people with less education, and people from countries higher in inequality, individualism, uncertainty, in long-term orientation, and restraint.^5^

## Discussion

The purpose of this study was to explore the relationship between country-level factors, valuing friendship, and people’s health, happiness, and subjective well-being. By analyzing data from the WVS, we captured a considerable number of individuals from a considerable number of countries from all around the world. The current report is the most comprehensive examination to date of how cultural factors affect the importance people place on friendships and how they benefit from them.

Older adults, women, people with higher levels of education, and people living in countries high in indulgence and lower income inequality placed a higher value on friendship. Several country-level factors—GDP, power distance, individualism, masculinity, uncertainty avoidance, and long-term orientation—did not predict how much value people placed on friendship. Similar to previous work, placing importance on friendships was strongly associated with better health, greater happiness, and higher levels of subjective well-being. Several individual- and country-level factors interacted with friendship importance to predict each outcome. Across all the outcomes, friendship importance was more strongly related to health and happiness among older adults, women, people with lower levels of education, and people living in individualistic cultures. A few additional moderators were also present, suggesting greater effects of friendship importance on the outcomes in countries higher in power distance, femininity, uncertainty, restraint, and long-term orientation. However, these moderation effects were not as consistent across the outcomes.

Although we took a largely exploratory approach in the current study, our findings have the potential to create a great deal of discussion and future research about how friendships, and social relationships more generally, vary across cultures. Naturally, our findings have many implications for theories in social and relationship sciences, including those that make hypotheses about the formation and maintenance of relationships (Rusbult, 1980), how the self varies across contexts—and the social implications of this variation (Kitayama et al., 2018), how economic and external stressors affect opportunities and outcomes of relationships (Ross et al., 2019), and even the social nature of emotions that can originate in friendships (Larson et al., 1986; van Kleef et al., 2016). In the current study, we provided important, basic descriptive information about how much—and some specific ways in which—cultures vary in the importance they place on friendships. As a result, researchers can begin to create more formalized models for why friendships are influential for health and well-being and the conditions under which these associations can be maximized (Hartup and Stevens, 1999; Sandstrom and Dunn, 2014). In the sections below, we provide a summary of our results, intentionally link the results to extant theory and research, and highlight the many remaining unknowns for how friendships—and the degree to which people value them—vary across cultures.

### Do Friendships and the Effect of Friendships Vary Across Individual- and Country-Level Factors?

We found that several individual- and country-level factors were significantly associated with variation in friendship importance. Some of these factors also interacted with valuing friendships to predict health and well-being. Below, we focus on discussing the factors with significant interactions.

#### Individual-Level Factors

Across cultures, women experienced greater well-being benefits when they rated friendships as important. Women’s friendships often consist of more intense emotional sharing and self-disclosure behavior compared to men’s friendships, and men’s friendships often involve more group activities and fewer expressions of affection and support (Wright, 1982). This may be why women value friendships more and yield greater benefits for their mental and physical well-being.

That older adults who valued friendships were happier suggests that placing high importance in social relationships can serve as a successful coping strategy that enhances well-being when encountering the adversity of older adulthood (Keller and Wood, 1989; Dykstra, 1995; Hutchinson et al., 2008; Cornwell and Waite, 2009; Chopik, 2017). A great deal of work is dedicated to how older adults fulfill their need to connect with others, which is a critical factor for preventing loneliness at this age (Charles, 2010; Masi et al., 2011). When older adults place low importance on friendship, they may be less likely to receive emotional and practical help from friends—leaving them exposed, with no buffers, to the negative emotions stemming from changes in their lives (e.g., declines in physical health). For younger adults, the contribution of friendship importance may not be as strong. Friendship importance may be less closely related to health and well-being given younger adults’ higher likelihood of deriving well-being from the achievement of information- and status-related goals in contrast to older adults’ focus on close relationships (Luong et al., 2011).

People who reported higher levels of education were happier, healthier, and reported higher levels of subjective well-being. However, people with lower levels of education benefited the most from placing a high importance on friendships. In other words, friendship importance partially compensated for many negative consequences associated with lower levels of education. There are a few possible explanations for the role of friendship importance in buffering against the negative effects of lower education on an individual’s quality of life. For instance, friend networks might provide additional social resources to people with lower levels of education, possibly narrowing the inequalities between them and highly educated individuals (Adler and Newman, 2002; Mirowsky and Ross, 2019).

#### Country-Level Factors

Valuing friendships was more strongly related to subjective well-being among people living in countries high in income inequality. Like the effects of education (for individuals), it could be that friendships buffer against negative societal pressures and conditions of living in a highly unequal society. However, ultimately, it is unclear why economic-related variables like education and income inequality modulate the benefits of social relationships on health and well-being. Future research can take a more holistic approach by examining the specific stressors that income inequality at the country-level causes for individuals and how friendship might ameliorate some of these stressors.

In general, we found that individualism predicted lower happiness and subjective well-being. However, placing higher importance on friendship was associated with particularly better health and happiness in countries high in individualism. Given that people from individualistic countries are more vulnerable to loneliness when they lack interactions with friends (Lykes and Kemmelmeier, 2014), it is not surprising that our study found a stronger association between friendship importance and health and well-being. The social arrangement of collectivistic cultures promotes interdependence and cherishes the well-being of the group (over the individual), which may result in obtaining more benefits from kin networks. In individualistic cultures, people might receive these benefits more from friendship networks. However, people in more individualistic countries tend to maintain high mobility within interpersonal relationships, value self-dependence, keep more personal space, and maintain weaker social ties (Markus and Kitayama, 1991; Trafimow et al., 1991; Kitayama et al., 1997)—for these people, valuing friends seem to buffer against the negative link between individualism and happiness/well-being.

Consistent with previous research, we found that uncertainty was related to worse health, lower happiness, and lower subjective well-being (Roll et al., 2015). However, friendship importance was more strongly related to subjective well-being in uncertain countries. Although country-level long-term orientation did not predict individual-level friendship importance, given that friends provide individuals with a sense of engagement and control over one’s life, valuing friendships still seems to buffer against the anxiety that arises from living in a country that is uncomfortable with uncertainty (Veiel and Baumann, 1992; Leary et al., 1995). Interestingly, people who value friendships were particularly healthy in countries with a long-term orientation. Although we are speculating, a country’s long-term orientation may impede well-being because it drives the country to implement changes that may be beneficial in the future but may not always translate to immediate improvements in individuals’ lives. It could be that people who value friendships are less affected by this long-term focus at the expense of immediate benefits for individuals.

Finally, indulgence predicted higher levels of health, happiness, and subjective well-being. Further, valuing friendships was particularly important for well-being in countries where indulgence was low (and restraints were higher). This aligns with previous research in which indulgence can be a strategy for upregulating positive emotions and reducing stress (Livingstone and Srivastava, 2012; Petersen et al., 2018). Friends are often a source of fun and pleasure, and among individuals who place importance on friends, they may yield more benefits in countries that are lower in indulgence. People living in countries higher in indulgence may not need to depend as closely on friendships to yield positive emotional benefits.

### Limitations and Future Directions

The current study had many strengths, as it employed a large sample of people from several different to examine the roles of friendship and culture on health and well-being. Nevertheless, there are limitations that should be addressed.

First, although our large sample enabled us to detect small effects and estimate effects with greater precision, the question of whether the effects are practically meaningful for individuals’ lives is worthy of discussion (Cohen, 1990; Funder and Ozer, 2019). This is especially the case for interactions between friendship importance and country-level factors, which tended to be the smallest in our study. Because this research was exploratory, it is possible that our large sample size resulted in some statistically significant—but not practically significant—findings. However, given that friendship was (and has been) an important predictor of health and well-being, it was important to examine how the contribution of friendship varied across different cultural contexts. In effect size terms, the differences between cultures were relatively small, suggesting that friendship is beneficial across many cultures. However, future work can examine the real-world significance of our effects, whether that be the effects of friendships interaction with a country’s economic or social standing or the number of years added to an individual’s life.

A second limitation was the way we assessed the importance of friendship and our outcome variables. More specifically, we used single-item indicators for most of our variables. Unfortunately, the WVS did not have any or sufficient information on the number of friends people had, the social activities they engaged in (and with whom), sufficient data on the amount of time spent with friends, or the actual quality of the participants’ friendships. Thus, we were only able to use a broad and crude indicator of friendship importance. Of course, knowing how much individuals think friendships are important is an informative measure—it likely gives some insight into how much they invest in the friendships in their lives. Further, more specific or nuanced measures [e.g., the number of “friends” (defined by participants) or quality of friendships] might differ according to individual-/country-level factors. Thus, a broader indicator of friendship investment with little ambiguity about its meaning may have been most appropriate for cross-cultural research. However, it would be important to have a multi-item indicator of friendship importance and directly compare it with other measures before making any conclusions. Future research should take a broader approach to the study of friendship by examining different measures of friendship investment and quality.

Related, the current study focused on a relatively narrow set of cultural indicators and did so in a largely exploratory fashion (see Footnote 3 for additional details). This approach also involved examining these cultural indicators at one static point in time. Worth noting, cultures and countries are not static entities and change considerably over time (Varnum and Kitayama, 2011; Varnum and Grossmann, 2017). For example, there is a great deal of evidence suggesting global increases in and shifts toward greater individualism (Grossmann and Varnum, 2015; Santos et al., 2017). Indeed, the relative weighting of the importance of friends versus family has even been considered to be at least a partial reflection of individualism (Santos et al., 2017). We did model year of data collection as a covariate in Footnote 5, but even these analyses fail to capture the dynamic nature of cultures, and using just one index of individualism (i.e., the Hofstede dimensions, which have received a great deal of criticism; Triandis et al., 1988; Schwartz, 1990; Talhelm, 2019) limited our ability in this regard. Future research should more thoughtfully model how cultural characteristics—and their psychological and health consequences—change and evolve over historical time (Chopik, 2020).

Finally, we hope that this report will provide useful information for other researchers in the formation of explicit hypotheses to test in future studies. Because of the lack of additional data available on valuing friendship and other potentially important variables, we were unable to test many of the mechanisms that we proposed might link friendship importance to health and well-being in certain cultures. For some cultures, valuing friendship might entail the exchange of instrumental support, which leads to better outcomes; for other cultures, it might entail the exchange of emotional support, which leads to better outcomes (Wilson et al., 1999; Merz and Huxhold, 2010; Rook, 2015). Further, these varying mechanisms might be dampened or enhanced based on additional cultural factors. Future researchers can use our preliminary findings to investigate why valuing friendships are associated with better outcomes in different contexts.

In pursuing these questions for future research, we would also like to advocate for methods and approaches that reduce researchers’ degrees of freedom when examining cultural differences in relational and psychosocial characteristics (Simmons et al., 2011; Roberts, 2015; Milfont and Klein, 2018; Vazire, 2018). This is especially true when approaching questions in such an exploratory way that we did here. For example, variation in the selection of cultural characteristics, variables measured or made available, analytic models, and interpretation criteria—many of which are arbitrary—can contribute to compromised reproduction of cultural differences that might undermine the science of cultural and relational differences. Unfortunately, we did not engage in these efforts in the current study but encourage others to do so. To this end, for both existing data sets and novel data collection efforts, preregistration and upfront justifications of these decision points can make for a more reproducible understanding of cultural differences in relational behavior (LeBel et al., 2017; Milfont and Klein, 2018; Haven and Van Grootel, 2019; Weston et al., 2019).

## Conclusion

This study examined the effects of valuing friendships on people’s health, happiness, and well-being among 323,200 individuals from 99 different countries around the world. The current study is the most comprehensive and diverse examination of friendships on health and well-being to date. Our findings suggest that valuing friendships is generally associated with better health, well-being, and happiness. In many cases, placing a high value on friendship was particularly important for health and well-being in settings typically associated with lower well-being (e.g., countries high in income inequality and individualism). Our findings highlight the importance of considering not only how much people value friendships but also the situating social relationships within broader individual and cultural contexts.

## Data Availability Statement

Publicly available datasets were analyzed in this study. This data can be found here: Data from the World Values Survey is publicly available for researchers. The study can be accessed via http://www.worldvaluessurvey.org/wvs.jsp.

## Ethics Statement

The analyses reported in this manuscript were deemed exempt from ethical oversight as it did not constitute the traditional type of human subjects research (MSU IRB#STUDY00002967).

## Author Contributions

PL and WC conceived the study. WC analyzed the data and created the tables and figures. PL, JO, KL, and WC drafted the manuscript and provided critical edits. All the authors contributed to the article and approved the submitted version.

## Conflict of Interest

The authors declare that the research was conducted in the absence of any commercial or financial relationships that could be construed as a potential conflict of interest.

## Footnotes

1 There are two additional items available in the WVS pertaining to friendships. One question was about living up to expectations put on individuals by friends (“Do you agree that you make a lot of effort to live up to what your friends expect?;” available for 59% of the sample) and a question about how often people spent time with friends (available for 17% of the sample). Because the former question is relatively ambiguous in its measurement (e.g., is it measuring a form of peer pressure, self-imposed pressure, or effort to invest in friendships), we ultimately decided not to include it in the current report. The latter question was available for too few people and countries that we elected to not include it here. However, in the interest of transparency, we are disclosing that these additional items related to friendships exist in the WVS.

2 Because many cultural indicators are often correlated with one another, we examined if any of the correlations between cultural variables were problematic and should not be entered simultaneously into the model. We found a strong negative correlation between power distance and individualism (*r* = −0.68). Upon running models sequentially, alternating the exclusion of these two variables, we found that the results did not differ at all, so we elected to include all of the variables in the model simultaneously.

3 There is seemingly an infinite number of variables on which cultures may differ. To reasonably present the main ways in which cultural variables might affect friendship importance, we focused on the Hofstede dimensions as they have been most clearly linked to cross-cultural variation in health and well-being and, to a degree, friendships. Nevertheless, there are many other taxonomies that could be chosen. Given reviewer feedback, we ran additional analyses predicting variation in friendship importance from social axiom characteristics (n = 37 countries available that overlapped with our current sample; Leung and Bond, 2004), self-construal (n = 28; Vignoles et al., 2016), relational mobility (n = 37; Thomson et al., 2018), Schwartz values (n = 63; Schwartz, 2006), GLOBE characteristics (n = 51; House et al., 2004), looseness–tightness (n = 27; Gelfand et al., 2011), human development (n = 94; UNDP, 2019a), population density (n = 94; UNDP, 2019b), and pathogen prevalence (n = 96; Murray and Schaller, 2010). The vast majority of these characteristics did not predict variation in friendship importance when tested in separate models: most social axioms (*p*s > 0.112), most forms of self-construal (*p*s > 0.092), relational mobility (*p* = 0.788), Schwartz values (*p*s > 0.06), GLOBE characteristics (*p*s > 0.08), looseness–tightness (*p*s = 0.629), human development (*p* = 0.440), population density (*p* = 0.440), and pathogen prevalence (*p* = 0.676). The two exceptions were that people from countries higher in social complexity (a social axiom; *r* = 0.525, *p* = 0.002) and countries higher in self-direction (a self-construal dimension; *r* = 0.43, *p* = 0.043) reported placing higher importance on friendships. We urge caution in interpreting these effects as there were relatively few countries available for the social axiom and self-construal analyses. The results are reported in Supplementary Tables 1–9.

4 Based on the recommendation of a reviewer, we examined the effects of cultural differences in response styles for our substantive results examining each outcome. Although it is difficult to conduct many formal examinations of response styles given our use of single-item indicators, we did run a number of supplementary analyses examining the impact of acquiescence on attenuating the results reported here. Following the recommended procedures (van Herk et al., 2004; Harzing, 2006), we calculated the proportion of individuals responding with extreme values [i.e., either 1 (for disacquiescence) or 4 (for acquiescence)] on our measure of friendship importance. The average acquiescence balance (acquiescence minus disacquiescence) for each country was used as a measure of acquiescence bias. This country level score ranged from 18.7 to 78.2% (*M* = 43.10, *SD* = 14.50). Entering acquiescence bias as a covariate in each model did not change the results in any way (i.e., there were minor/small variations in the estimates but none that changed the significance or interpretation of the effects). This could be partially due to acquiescence biases being largely unrelated to happiness (*p* = 0.28) and subjective well-being (*p* = 0.81). Acquiescence bias was associated with physical health (*t* = 2.58, *p* = 0.01) such that countries choosing more extreme values on the friendship variable reported better health. However, even in the context of this significant effect, the other main effects and interactions were the same in their significance and interpretation. Although we were limited in the degree to which we could assess the effects of response style biases, we encourage future researchers to more formally examine sources of this variation and how this variation might affect conclusions about cultural differences in social and psychological constructs.

5 A helpful reviewer suggested that we take advantage of the multiwave assessments and large sample size of the WVS. Specifically, we controlled for wave of data collection and found that people in more recent waves valued friendships more (linear effect of wave; *r* = 0.035, *p* < 0.001), were healthier (*r* = 0.068, *p* < 0.001), were happier (*r* = 0.066, *p* < 0.001), and reported higher life satisfaction (*r* = 0.057, *p* < 0.001); none of our substantive findings changed (although some marginally significant effects became non-significant). They also recommended that we use a cross-validation approach in which the data set is split in half to see how reliable the findings were (Weston et al., 2019). Although this was suggested after the main analyses were already done, we still thought it was a good idea and provide some test of how robust the findings are. Thus, we split the sample in half and re-ran all of our analyses on both halves. We found the results from Table 1, were reproduced across the two smaller halves. For the analyses in Tables 2–4, most of the moderation effects were reproduced across the analyses, with just a few exceptions, specifically, the power distance and long-term orientation moderation effects (for health), the power distance moderation effect (for happiness), and the long-term orientation moderation effect (for subjective well-being). Thus, these particular moderation effects should be viewed with some skepticism given that they were not reproduced across the two random halves of the data set.
