# Supplementary material for: Friendship Importance Around the World: Links to Cultural Factors, Health, and Well-Being
Source: Front Psychol. 2021 Jan 18;11:570839. doi: 10.3389/fpsyg.2020.570839 (PMC7848226; doi:10.3389/fpsyg.2020.570839)
Supplement: Supplementary file 1 [file Data_Sheet_1.docx]

| Supplementary Table 1. Multi-level model predicting friendship importance from social axioms | | | |  |  |  |  |
| --- | --- | --- | --- | --- | --- | --- | --- |
|  | *b* | *SE* | *t* | *p* | LB | UB | *r* |
| Age | -0.002 | 0.000 | -19.895 | 0.000 | -0.003 | -0.002 | 0.054 |
| Gender | 0.003 | 0.002 | 1.430 | 0.153 | -0.001 | 0.006 | 0.004 |
| Education | 0.024 | 0.001 | 27.056 | 0.000 | 0.022 | 0.026 | 0.074 |
| Social Cynicism | -0.306 | 0.187 | -1.637 | 0.112 | -0.689 | 0.076 | 0.287 |
| Social Complexity | 0.813 | 0.241 | 3.374 | 0.002 | 0.321 | 1.306 | 0.525 |
| Reward for Application | -0.110 | 0.182 | -0.605 | 0.550 | -0.482 | 0.262 | 0.110 |
| Religiosity | 0.048 | 0.115 | 0.420 | 0.678 | -0.187 | 0.283 | 0.076 |
| Fate Control | 0.145 | 0.175 | 0.827 | 0.415 | -0.213 | 0.503 | 0.150 |

| Supplementary Table 2. Multi-level model predicting friendship importance from self-construal characteristics | | | |  |  |  |  |
| --- | --- | --- | --- | --- | --- | --- | --- |
|  | *b* | *SE* | *t* | *p* | LB | UB | *r* |
| Age | -0.002 | 0.000 | -14.719 | 0.000 | -0.002 | -0.002 | 0.045 |
| Gender | 0.002 | 0.002 | 0.808 | 0.419 | -0.002 | 0.006 | 0.002 |
| Education | 0.027 | 0.001 | 27.203 | 0.000 | 0.025 | 0.029 | 0.083 |
| Diff v. Sim | -3.802 | 1.849 | -2.056 | 0.053 | -7.659 | 0.055 | 0.417 |
| Self-contain v. Connection | -0.278 | 0.517 | -0.538 | 0.596 | -1.356 | 0.800 | 0.119 |
| Self-direction v. Reception | 1.516 | 0.701 | 2.162 | 0.043 | 0.054 | 2.979 | 0.434 |
| Self-reliance v. Dependence | 6.103 | 3.444 | 1.772 | 0.092 | -1.078 | 13.285 | 0.368 |
| Consistency v. Variability | -2.309 | 1.309 | -1.764 | 0.093 | -5.038 | 0.420 | 0.366 |
| Self-expression v. Harmony | 6.010 | 3.183 | 1.888 | 0.074 | -0.628 | 12.648 | 0.388 |
| Self-interest v. Commitment | 0.338 | 0.430 | 0.785 | 0.442 | -0.560 | 1.235 | 0.173 |

| Supplementary Table 3. Multi-level model predicting friendship importance from relational mobility | | | | |  |  |  |
| --- | --- | --- | --- | --- | --- | --- | --- |
|  | *b* | *SE* | *t* | *p* | LB | UB | *r* |
| Age | -0.002 | 0.000 | -12.362 | 0.000 | -0.002 | -0.001 | 0.034 |
| Gender | 0.009 | 0.002 | 4.686 | 0.000 | 0.005 | 0.013 | 0.013 |
| Education | 0.026 | 0.001 | 28.836 | 0.000 | 0.024 | 0.028 | 0.080 |
| Relational Mobility | -0.041 | 0.152 | -0.271 | 0.788 | -0.349 | 0.267 | 0.046 |

| Supplementary Table 4. Multi-level model predicting friendship importance From Schwartz's cultural values | | | | |  |  |  |
| --- | --- | --- | --- | --- | --- | --- | --- |
|  | *b* | *SE* | *t* | *p* | LB | UB | *r* |
| Age | -0.002 | 0.000 | -21.468 | < .001 | -0.002 | -0.002 | 0.045 |
| Gender | -0.002 | 0.001 | -1.062 | 0.288 | -0.004 | 0.001 | 0.002 |
| Education | 0.029 | 0.001 | 40.954 | < .001 | 0.028 | 0.031 | 0.086 |
| Harmony | -0.108 | 0.153 | -0.708 | 0.482 | -0.415 | 0.198 | 0.096 |
| Embeddedness | 0.346 | 0.267 | 1.296 | 0.200 | -0.189 | 0.881 | 0.174 |
| Hierarchy | -0.103 | 0.084 | -1.226 | 0.226 | -0.270 | 0.065 | 0.164 |
| Mastery | -0.082 | 0.249 | -0.331 | 0.742 | -0.581 | 0.417 | 0.045 |
| Affective Autonomy | 0.238 | 0.124 | 1.923 | 0.060 | -0.010 | 0.486 | 0.253 |
| Intellectual Autonomy | 0.126 | 0.184 | 0.685 | 0.496 | -0.243 | 0.494 | 0.093 |
| Egalitarianism | 0.176 | 0.139 | 1.271 | 0.209 | -0.102 | 0.455 | 0.170 |

| Supplementary Table 5. Multi-level model predicting friendship importance from GLOBE Characteristics | | | | |  |  |  |
| --- | --- | --- | --- | --- | --- | --- | --- |
|  | *b* | *SE* | *t* | *p* | LB | UB | *r* |
| Age | -0.002 | 0.000 | -16.559 | < .001 | -0.002 | -0.002 | 0.038 |
| Gender | 0.003 | 0.002 | 2.100 | 0.036 | 0.000 | 0.007 | 0.005 |
| Education | 0.027 | 0.001 | 35.522 | < .001 | 0.026 | 0.029 | 0.082 |
| Uncertainty Avoidance Societal Practices | 0.057 | 0.091 | 0.632 | 0.532 | -0.128 | 0.242 | 0.113 |
| Future Orientation Societal Practices | 0.031 | 0.103 | 0.304 | 0.763 | -0.179 | 0.241 | 0.054 |
| Power Distance Societal Practices | -0.076 | 0.140 | -0.543 | 0.591 | -0.361 | 0.209 | 0.097 |
| Collectivism I Societal Practices (Institutional Collectivism) | 0.010 | 0.102 | 0.101 | 0.920 | -0.198 | 0.219 | 0.018 |
| Humane Orientation Societal Practices | -0.021 | 0.079 | -0.267 | 0.791 | -0.181 | 0.139 | 0.048 |
| Performance Orientation Societal Practices | -0.180 | 0.100 | -1.808 | 0.080 | -0.383 | 0.023 | 0.308 |
| Collectivism II Societal Practices (In-group Collectivism) | -0.091 | 0.075 | -1.207 | 0.237 | -0.244 | 0.063 | 0.211 |
| Gender Egalitarianism Societal Practices | -0.128 | 0.102 | -1.259 | 0.217 | -0.336 | 0.079 | 0.220 |
| Assertiveness Societal Practices | 0.055 | 0.101 | 0.548 | 0.587 | -0.150 | 0.261 | 0.098 |
| Uncertainty Avoidance Societal Values | -0.073 | 0.100 | -0.729 | 0.472 | -0.276 | 0.131 | 0.130 |
| Future Orientation Societal Values | 0.008 | 0.122 | 0.070 | 0.945 | -0.239 | 0.256 | 0.012 |
| Power Distance Societal Values | 0.027 | 0.126 | 0.216 | 0.831 | -0.229 | 0.283 | 0.039 |
| Collectivism I Societal Values (Institutional Collectivism) | -0.019 | 0.088 | -0.215 | 0.831 | -0.199 | 0.161 | 0.039 |
| Human Orientation Societal Values | 0.180 | 0.121 | 1.490 | 0.146 | -0.066 | 0.426 | 0.258 |
| Performance Orientation Societal Values | -0.053 | 0.093 | -0.570 | 0.573 | -0.243 | 0.137 | 0.102 |
| Collectivism II Societal Values (In-group Collectivism) | 0.172 | 0.106 | 1.622 | 0.115 | -0.044 | 0.388 | 0.279 |
| Gender Egalitarianism Societal Values | -0.097 | 0.098 | -0.989 | 0.330 | -0.296 | 0.103 | 0.174 |
| Assertiveness Societal Values | 0.021 | 0.047 | 0.439 | 0.664 | -0.076 | 0.117 | 0.079 |

| Supplementary Table 6. Multi-level model predicting friendship importance from tightness | | | | |  | |  | |  | |  |
| --- | --- | --- | --- | --- | --- | --- | --- | --- | --- | --- | --- |
|  | *b* | *SE* | *t* | *p* | | LB | | UB | | *r* | |
| Age | -0.002 | 0.000 | -15.979 | 0.000 | | -0.002 | | -0.002 | | 0.050 | |
| Gender | 0.012 | 0.002 | 5.846 | 0.000 | | 0.008 | | 0.016 | | 0.018 | |
| Education | 0.029 | 0.001 | 28.083 | 0.000 | | 0.027 | | 0.031 | | 0.087 | |
| Tightness | -0.006 | 0.011 | -0.489 | 0.629 | | -0.029 | | 0.018 | | 0.099 | |

| Supplementary Table 7. Multi-level model predicting friendship importance from the human development index | | | | | |  | |  | |  | |
| --- | --- | --- | --- | --- | --- | --- | --- | --- | --- | --- | --- |
|  | *b* | *SE* | *t* | *p* | LB | | UB | | *r* | |  |
| Age | -0.002 | 0.000 | -24.099 | 0.000 | -0.002 | | -0.002 | | 0.046 | |  |
| Gender | -0.011 | 0.001 | -8.070 | 0.000 | -0.013 | | -0.008 | | 0.015 | |  |
| Education | 0.027 | 0.001 | 41.848 | 0.000 | 0.026 | | 0.028 | | 0.080 | |  |
| Human Development | 0.135 | 0.175 | 0.776 | 0.440 | -0.211 | | 0.482 | | 0.081 | |  |

| Supplementary Table 8. Multi-level model predicting friendship importance from population density | | | | |  |  |  |
| --- | --- | --- | --- | --- | --- | --- | --- |
|  | *b* | *SE* | *t* | *p* | LB | UB | *r* |
| Age | -0.002 | 0.000 | -24.367 | 0.000 | -0.002 | -0.002 | 0.046 |
| Gender | -0.010 | 0.001 | -7.731 | 0.000 | -0.013 | -0.008 | 0.015 |
| Education | 0.027 | 0.001 | 41.983 | 0.000 | 0.026 | 0.028 | 0.079 |
| Population Density | 0.000 | 0.000 | -0.158 | 0.875 | 0.000 | 0.000 | 0.017 |

| Supplementary Table 9. Multi-level model predicting friendship importance from pathogen prevalence | | | | |  | |  | |  | |  |
| --- | --- | --- | --- | --- | --- | --- | --- | --- | --- | --- | --- |
|  | *b* | *SE* | *t* | *p* | | LB | | UB | | *r* | |
| Age | -0.002 | 0.000 | -24.198 | 0.000 | | -0.002 | | -0.002 | | 0.045 | |
| Gender | -0.010 | 0.001 | -7.777 | 0.000 | | -0.013 | | -0.008 | | 0.015 | |
| Education | 0.027 | 0.001 | 42.878 | 0.000 | | 0.026 | | 0.029 | | 0.080 | |
| Pathogen Prevalence | -0.015 | 0.035 | -0.419 | 0.676 | | -0.085 | | 0.055 | | 0.043 | |
